# Risk for International Importations of Variant SARS-CoV-2 Originating in the United Kingdom

**DOI:** 10.3201/eid2705.210050

**Published:** 2021-05

**Authors:** Zhanwei Du, Lin Wang, Bingyi Yang, Sheikh Taslim Ali, Tim K. Tsang, Songwei Shan, Peng Wu, Eric H.Y. Lau, Benjamin J. Cowling, Lauren Ancel Meyers

**Affiliations:** WHO Collaborating Centre for Infectious Disease Epidemiology and Control, University of Hong Kong, Hong Kong, China (Z. Du, B. Yang, S.T. Ali, T.K. Tsang, S. Shan, P. Wu, E.H.Y. Lau, B.J. Cowling);; Laboratory of Data Discovery for Health, Hong Kong (Z. Du, S.T. Ali, S. Shan, P. Wu, E.H.Y. Lau, B.J. Cowling);; University of Cambridge, Cambridge, UK (L. Wang);; The University of Texas at Austin, Austin, Texas, USA (L.A. Meyers);; Santa Fe Institute, Santa Fe, New Mexico, USA (L.A. Meyers)

**Keywords:** 2019 novel coronavirus disease, coronavirus disease, COVID-19, severe acute respiratory syndrome coronavirus 2, SARS-CoV-2, viruses, respiratory infections, zoonoses, variant, epidemiology, importation, United Kingdom

## Abstract

A fast-spreading severe acute respiratory syndrome coronavirus 2 variant identified in the United Kingdom in December 2020 has raised international alarm. We analyzed data from 15 countries and estimated that the chance that this variant was imported into these countries by travelers from the United Kingdom by December 7 is >50%.

The United Kingdom has detected a variant of severe acute respiratory syndrome coronavirus 2 (SARS-CoV-2), the causative agent coronavirus disease (COVID-19), from samples initially collected in Kent on September 20 and London on September 21, 2020 (*1*). The variant was associated with increased transmissibility and includes deletions at amino acid sites 69 and 70 of the spike protein (*2*). In mid-December, the UK government tightened measures in London and southeastern England to mitigate transmission of the fast-spreading virus variant (*3*). On January 5, 2021, England initiated a national lockdown that included closing all schools and nonessential businesses until mid-February (*4*). By December 20, restrictions for travelers from the United Kingdom had been implemented by ≈40 countries (*5*). The new variant (501Y) has subsequently been reported worldwide, including in the United States (*6*), Spain, Sweden, and France, and might be spreading without detection in countries with limited virus sequencing capacity (*5*).

Using data from 15 countries, we estimated the probability that travelers from the United Kingdom introduced this 501Y variant into each of the countries and estimated the extent of local transmission. Our estimations were based on the changing proportion of infections caused by the 501Y variant identified in the United Kingdom (*2*) and population mobility from the United Kingdom to each country, determined from Facebook Data for Good (https://dataforgood.fb.com). The highest risk for importation from September 22 through December 7, 2020, was in Ireland. By October 22 (a month after the variant was first detected in the United Kingdom), the chance that 10 of the 15 countries would receive 1 imported case from the United Kingdom was at least 50% (Figure), except for Romania, Portugal, Cyprus, India, and the United States, although by November 1, this risk threshold was exceeded for all of these countries.

**Figure:**
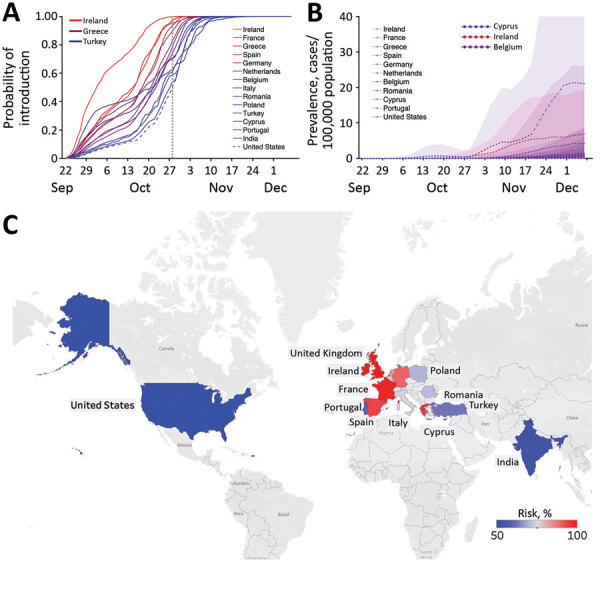
Estimated risks for introduction of the 501Y variant of severe acute respiratory syndrome coronavirus 2 (SARS-CoV-2) from the United Kingdom to 15 other countries before December 7, 2020. A) Probability that >1 person infected with this SARS-CoV-2 variant arrived at the target country from the United Kingdom by the date indicated on the x-axis, based on Facebook mobility data (https://dataforgood.fb.com). The dotted gray vertical line indicates October 28, 2020, the date when the introduction risk for the United States surpassed 50%; line colors correspond to the relative risk for importations as of that date. B) Estimated daily prevalence of the 501Y variant of SARS-CoV-2 in 11 countries between September 22 and December 7, 2020, assuming that the variant is , which means 50% more transmissible than the 501N variant (*11*). Points and bands indicate means and SDs based on 100 simulations. C) Probability of >1 variant importation by October 28, 2020. Grey indicates countries/regions where mobility data were not available.

Using COVID-19 hospital admission data, we further estimated the local prevalence of the 501Y variant in 11 of the 15 countries, assuming that the 501Y variant is 50% more transmissible than the circulating 501N strain (Figure). The variant seems to have ascended fastest in Ireland before slowing in mid-November and is expected to be spreading rapidly in many of the other countries. As of December 7, the expected prevalence of the variant and the expected proportion of coronavirus disease cases were highest in Cyprus (prevalence 13 cases, 95% CI 0–79 cases/100,000 population; proportion 6% of cases, 95% CI 0–38% of cases) (Figure; Appendix Figures 1, 2).

These projections suggest that countries with substantial population movement from the United Kingdom were likely to harbor cases of the 501Y variant by late October 2020. Our conclusions were based on several key assumptions. The mobility data, which include ≈3 million trips from the United Kingdom to the 15 countries we analyzed, might be demographically biased by the user profile of Facebook, a major social media company with ≈2.8 billion monthly active users in the fourth quarter of 2020 (*7*). We assume that all introductions during this early period occurred via asymptomatic travelers from the United Kingdom and ignore possible importations from other countries or by symptomatic case-patients traveling to seek healthcare. A sensitivity analysis suggests that these assumptions may cause a downward bias in the estimated rates of global expansion (Appendix Figure 3). Furthermore, we assume a 10-day lag between infection and hospitalization on the basis of estimates from the United States (*8*) and Europe (*9*) and estimate the daily prevalence of the 501Y variant by using the method introduced in (*2*), under the assumptions that the 2 variants (501Y and 501N) share the same natural history (*2*) and symptomatic proportion (*10*,*11*). Should future studies reveal substantial epidemiologic differences between the variant and wildtype, then these estimates can be readily updated by using the full equations provided in (*2*).

Appendix Supplemental methods and results for study of risk for international importations of variant SARS-CoV-2 originating in the United Kingdom.
