# Supplementary material for: Risk for International Importations of Variant SARS-CoV-2 Originating in the United Kingdom
Source: Emerg Infect Dis. 2021 May;27(5):1527–9. doi: 10.3201/eid2705.210050 (PMC8084518; doi:10.3201/eid2705.210050)
Supplement: Appendix — Supplemental methods and results for study of risk for international importations of variant SARS-CoV-2 originating in the United Kingdom. [file 21-0050-Techapp-s1.pdf]

Article DOI: https://doi.org/10.3201/eid2705.210050

Risk for International Importations of Variant SARS-CoV-2 Originating in the United Kingdom

Appendix

Data

To estimate the daily number of passengers traveling between the UK and other countries, we obtained daily mobility data from Facebook Data for Good (*1*). Based on geolocation data from Facebook mobile users that enable the 'location history' feature, Facebook constructed anonymized origin–destination mobility matrices between countries. However, Facebook only reports mobility flows from the UK to other countries when the daily number of recorded trips is at least 1000. We excluded countries from our analysis that had over 30% days between September 22 and October 21, 2020 falling below this threshold. For the countries we analyzed, we assumed a random number of trips (uniformly distributed between 0 and 1000) on each day of missing data. The data for those countries include ~3 million trips originating in the UK between September 22 and December 14, 2020.

Methods

Risk of COVID-19 variant introduction via infected travelers from the UK

To estimate the probability at least one case has been introduced from the UK into a given country, we first estimate the prevalence of pre-symptomatic and asymptomatic cases in the UK and then use mobility data to estimate the likelihood of introductions.

Our notation and parameter values are provided in Appendix Table. Briefly, we assume that a fraction p_{sym} of cases eventually develop symptoms and the remaining 1- p_{sym} remain asymptomatic throughout the course of their infection. The total periods of infection for asymptomatic and symptomatic cases are $D_{\mathrm{inf,a}}$ and $D_{\mathrm{inf,s}}$ days, respectively, with symptomatic cases developing symptoms following a D_{presym} day incubation period. A proportion is denoted r

of symptomatic cases require hospitalization and enter the hospital D_{h} days following infection. We use dH_t^{UK} and ω_t to denote the number of new COVID-19 hospital admissions and the proportion of the new variant among all sequenced SARS-CoV-2 samples in the UK on day *t*, respectively. We assume that the numbers of new pre-symptomatic and asymptomatic variant-caused infections in the UK at time *t* are given by

$$dI_{\mathrm{presym},t}^{\mathrm{UK}} = \frac{dH_{t+D_{\mathrm{h}}}^{\mathrm{UK}}\omega_t}{r}$$

$$dI_{\mathrm{asym},t}^{\mathrm{UK}} = dI_{\mathrm{presym},t}^{\mathrm{UK}}\left(\frac{1-p_{\mathrm{sym}}}{p_{\mathrm{sym}}}\right).$$

Then, the numbers of pre-symptomatic and asymptomatic cases in the UK at time *t* are given by

$$I_{\mathrm{presym},t}^{\mathrm{UK}} = \sum_{i=t-D_{\mathrm{presym}}}^{t-1} dI_{\mathrm{presym},i}^{\mathrm{UK}}$$

$$I_{\mathrm{asym},t}^{\mathrm{UK}} = \sum_{i=t-D_{\mathrm{inf,a}}}^{t-1} dI_{\mathrm{asym},i}^{\mathrm{UK}}$$

The prevalence of asymptomatic cases infected by the variant as a proportion of the UK population is given by

$$\xi_t^{\mathrm{UK}} = \frac{I_{\mathrm{presym},t}^{\mathrm{UK}} + I_{\mathrm{asym},t}^{\mathrm{UK}}}{N_{\mathrm{UK}}}$$

where N_{UK} is the population size of the UK.

Assuming that travelers from the UK to other countries are infected with the variant according to the overall prevalence of pre-symptomatic and asymptomatic cases (assuming symptomatic cases will not travel), the rate of introductions from the UK to country *c* on day *t* is approximated by

$$\gamma_t^c = \xi_t^{\mathrm{UK}} \cdot \Omega_t^c,$$

where Ω_t^c is the daily number of travelers from the UK to country *c*.

Assuming that the introduction of variant cases from the UK to each country *c* is essentially a non-homogeneous Poisson process (*2*–*4*), we estimate the probability of at least one introduction by time *t* (starting at time t_0) using

$$1 - \exp(-\sum_{i=t_0}^{t} \gamma_i^c).$$

Estimating the prevalence of the variant in each country

We assume the variant and wildtype share the same generation time (*5*). Let dH_t^c denote the number of new COVID-19 hospitalizations in country *c* on day *t*. We assume that the number of new symptomatic and asymptomatic COVID-19 cases in country *c* on day *t* are given by

$$dI_{\mathrm{presym},t}^c = \frac{dH_{t+D_\mathrm{h}}^c}{r}$$

$$dI_{\mathrm{asym},t}^c = dI_{\mathrm{presym},t}^c \left(\frac{1 - p_\mathrm{sym}}{p_\mathrm{sym}} \right).$$

Let σ denote the ratio between the reproduction number of the 501Y variant and the reproduction number of the 501N variant. Then, the incidence of the 501Y and 501N variants are given by

$$dI_{\mathrm{Y},t}^c = (dI_{\mathrm{presym},t}^c + dI_{\mathrm{asym},t}^c) \frac{\sigma I_{\mathrm{Y},t-1}^c}{\sigma I_{\mathrm{Y},t-1}^c + I_{\mathrm{N},t-1}^c}$$

$$dI_{\mathrm{N},t}^c = (dI_{\mathrm{presym},t}^c + dI_{\mathrm{asym},t}^c) \frac{I_{\mathrm{N},t-1}^c}{\sigma I_{\mathrm{Y},t-1}^c + I_{\mathrm{N},t-1}^c}.$$

We estimate the prevalence of the 501Y and 501N variants as a proportion of the population, as given by

$$I_{\mathrm{Y},t}^c = \frac{1}{N_c} \left(\sum_{i=t-D_{\mathrm{inf,s}}}^{t-1} dI_{\mathrm{Y},i}^c p_\mathrm{sym} + \sum_{i=t-D_{\mathrm{inf,a}}}^{t-1} dI_{\mathrm{Y},i}^c (1 - p_\mathrm{sym}) \right)$$

$$I_{\mathrm{N},t}^c = \frac{1}{N_c} \left(\sum_{i=t-D_{\mathrm{inf,s}}}^{t-1} dI_{\mathrm{N},i}^c p_\mathrm{sym} + \sum_{i=t-D_{\mathrm{inf,a}}}^{t-1} dI_{\mathrm{N},i}^c (1 - p_\mathrm{sym}) \right).$$

At the start of each simulation, we assume that all cases on September 22, 2020 are the wildtype (501N). That is, $I^c_{\mathrm{Y},0} = 0$ and $I^c_{\mathrm{N},0} = dI^c_{\mathrm{presym},0} + dI^c_{\mathrm{asym},0}$. Variant (501Y) cases are then introduced according to our travel model. Prior to the first introduction of the variant into country *c*, we draw a poisson random variable with a mean of γ^c_t each day *t* (representing the number of asymptomatic and presymptomatic cases of 501Y traveling from the UK to *c*). At the first instance of a nonzero number of importations into country *c*, we set $dI^c_{\mathrm{Y},t} = \gamma^c_t$ and do not consider subsequent importations into *c*.

Sensitivity Analyses

Appendix Figures 1 and 2 provide a sensitivity analysis with respect to the relative transmission rate of the variant, with σ ranging from 25% to 75% more transmissible than the wildtype (Appendix Figures 1 and 2). Appendix Figure 3 provides a sensitivity analysis in which we simultaneously change the following two assumptions, both of which accelerate our estimates for importation times: (i) non-hospitalized symptomatic cases are allowed to travel and (ii) the mobility values are roughly doubled (scaled by 741/419) to account for the FaceBook data sample size (419 million regular users (*6*) out of a Europe population of 741 million (*7*) in 2020).

References

1. Facebook Data for Good. [cited 2021 Jan 2]. https://dataforgood.fb.com

2. Wang L, Wu JT. Characterizing the dynamics underlying global spread of epidemics. Nat Commun. 2018;9:218. PubMed https://doi.org/10.1038/s41467-017-02344-z

3. Scalia Tomba G, Wallinga J. A simple explanation for the low impact of border control as a countermeasure to the spread of an infectious disease. Math Biosci. 2008;214:70–2. PubMed https://doi.org/10.1016/j.mbs.2008.02.009

4. Wu JT, Leung K, Leung GM. Nowcasting and forecasting the potential domestic and international spread of the 2019-nCoV outbreak originating in Wuhan, China: a modelling study. Lancet. 2020;395:689–97. PubMed https://doi.org/10.1016/S0140-6736(20)30260-9

5. Leung K, Shum MHH, Leung GM, Lam TTY, Wu JT. Early transmissibility assessment of the N501Y mutant strains of SARS-CoV-2 in the United Kingdom, October to November 2020. Euro Surveill. 2021;26:2002106. PubMed https://doi.org/10.2807/1560-7917.ES.2020.26.1.2002106

6. Facebook: Europe monthly active users by quarter 2020 [Internet]. [cited 2021 Mar 6]. Available from: https://www.statista.com/statistics/745400/facebook-europe-mau-by-quarter

7. European Union (EU) - total population 2020 [Internet]. [cited 2021 Mar 6]. Available from: https://www.statista.com/statistics/253372/total-population-of-the-european-union-eu

8. Gudbjartsson DF, Helgason A, Jonsson H, Magnusson OT, Melsted P, Norddahl GL, et al. Spread of SARS-CoV-2 in the Icelandic Population. N Engl J Med. 2020;382:2302–15. PubMed https://doi.org/10.1056/NEJMoa2006100

9. King's College London. No evidence of change in symptoms from new coronavirus variant. King's College London. 2021 [cited 2021 Mar 3]. https://www.kcl.ac.uk/news/no-evidence-change-symptoms-new-coronavirus-variant

10. Davies NG, Abbott S, Barnard RC, Jarvis CI, Kucharski AJ, Munday JD, et al. Estimated transmissibility and impact of SARS-CoV-2 lineage B.1.1.7 in England. Science. 2021 Mar 3 [cited 2021 Mar 4]. https://science.sciencemag.org/content/early/2021/03/03/science.abg3055

11. Official UK Coronavirus Dashboard [cited 2020 Dec 29]. https://coronavirus.data.gov.uk/details/healthcare

12. Coronavirus (COVID-19) Hospitalizations [cited 2021 Jan 3]. https://ourworldindata.org/covid-hospitalizations

13. CDC. Estimated Disease Burden of COVID-19. 2020 [cited 2020 Dec 28]. https://www.cdc.gov/coronavirus/2019-ncov/cases-updates/burden.html

14. Backer JA, Klinkenberg D, Wallinga J. Incubation period of 2019 novel coronavirus (2019-nCoV) infections among travellers from Wuhan, China, 20-28 January 2020. Euro Surveill. 2020;25:2000062. PubMed https://doi.org/10.2807/1560-7917.ES.2020.25.5.2000062

15. Aleta A, Martín-Corral D, Pastore Y, Piontti A, Ajelli M, Litvinova M, et al. Modelling the impact of testing, contact tracing and household quarantine on second waves of COVID-19. Nat Hum Behav. 2020 Aug 5. http://dx.doi.org/10.1038/s41562-020-0931-9 https://doi.org/10.1038/s41562-020-0931-9

16. ISARIC. International Severe Acute Respiratory and Emerging Infections Consortium (ISARIC). COVID-19 Report: 19 [cited 2021 Feb 1]. https://media.tghn.org/medialibrary/2020/05/ISARIC_Data_Platform_COVID-19_Report_19MAY20.pdf

17. Park N. Population estimates for the UK, England and Wales, Scotland and Northern Ireland - Office for National Statistics. Office for National Statistics; 2020 [cited 2020 Dec 29]. https://www.ons.gov.uk/peoplepopulationandcommunity/populationandmigration/populationestimates/bulletins/annualmidyearpopulationestimates/mid2019estimates

18. World Population Prospects - Population Division - United Nations [cited 2021 Feb 3]. https://population.un.org/wpp/Download/Standard/Population/

19. Report 42 - Transmission of SARS-CoV-2 Lineage B.1.1.7 in England: insights from linking epidemiological and genetic data [cited 2021 Jan 3]. https://www.imperial.ac.uk/mrc-global-infectious-disease-analysis/covid-19/report-42-sars-cov-2-variant

Appendix Table. Model Parameters and Data Sources

Symbol	Description	Values	Source (Reference)
Ω_t^c	Number of travelers from the UK to country *c* at time *t*	Daily mobility	Facebook Data for Good (*1*)
p_{sym}	Proportion of infections that are symptomatic	57%	(*8*),<br>assuming 501Y and 501N have the same symptomatic proportion (*9,10*)
dH_t^{UK}	Number of COVID-19 hospital admissions in the UK at time *t*	Daily admissions	(*11,12*)
$dI_{\mathrm{presym},t}^{\mathrm{UK}}$	Number of new pre-symptomatic variant infections in the UK at time *t*	Daily cases	Estimated
$dI_{\mathrm{asym},t}^{\mathrm{UK}}$	Number of new asymptomatic variant infections in the UK at time *t*	Daily cases	Estimated
ξ_t^{UK}	Prevalence of asymptomatic variant cases as a percentage of the UK population at time *t*	Daily prevalence	Estimated
γ_t^c	Rate of variant introductions from the UK to country *c* on day *t*	Daily rate	Estimated
dH_t^c	Number of COVID-19 hospital admissions in country *c* at time *t*	Daily admissions	(*11,12*)
$dI_{\mathrm{presym},t}^c$	Number of new pre-symptomatic variant infections in country *c* at time *t*	Daily cases	Estimated
$dI_{\mathrm{asym},t}^c$	Number of new asymptomatic variant infections in country *c* at time *t*	Daily cases	Estimated
r	Proportion of symptomatic cases that are hospitalized	3.4/77	(*13*)
ω_t	Proportion of the 501Y variant (with deletions at amino acid sites 69/70 of the S protein) among new SARS-CoV-2 cases at time *t*	Proportion of sequenced SARS-CoV-2 specimens in UK (weekly data)	(5)
D_{h}	Expected delay from infection to hospital admission	10 d	5 d from infection to symptom onset (*14*); 5 d from symptom onset to hospital admission (*15,16*)
D_{presym}	Expected incubation period between infection and symptom onset for symptomatic cases	5 d	(*14*)
$D_{\mathrm{inf,s}}$	Expected time from infection to recovery, for symptomatic cases	11 d	Sum of expected 5-d incubation (*14*) and expected 6-d symptomatic (*15*) period
$D_{\mathrm{inf,a}}$	Expected time from infection to recovery, for asymptomatic cases	11 d	(*15*)
N_{UK}	Population of United Kingdom	66,796,807 (2019)	(*17*)

Symbol	Description	Values	Source (Reference)
N_c	Population of country *c*	2019 populations	(*18*)
σ	Ratio of the reproduction numbers for the 501Y versus 501N variant	1.25, 1.5, and 1.75	Assumed based on (*5,10,19*)

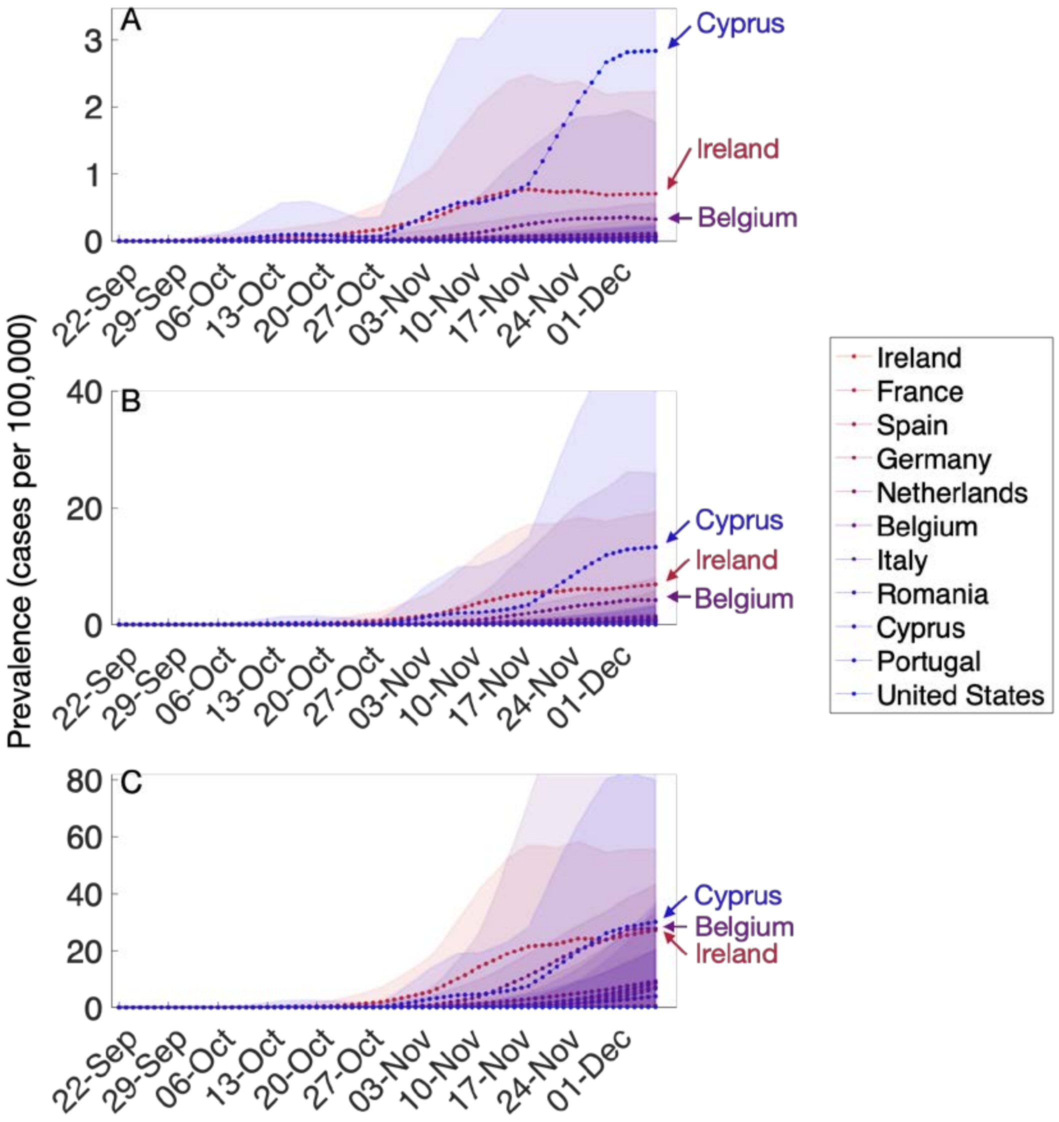

Appendix Figure 1. Estimated daily prevalence of the 501Y variant of SARS-CoV-2 in 15 countries between September 22 and December 7, 2020, assuming the variant is σ = (A) 25%, (B) 50%, and (C) 75% more transmissible than 501N. Points and bars indicate means and standard deviations based on 100 simulations. Countries with highest estimated prevalence on December 7th are labeled.

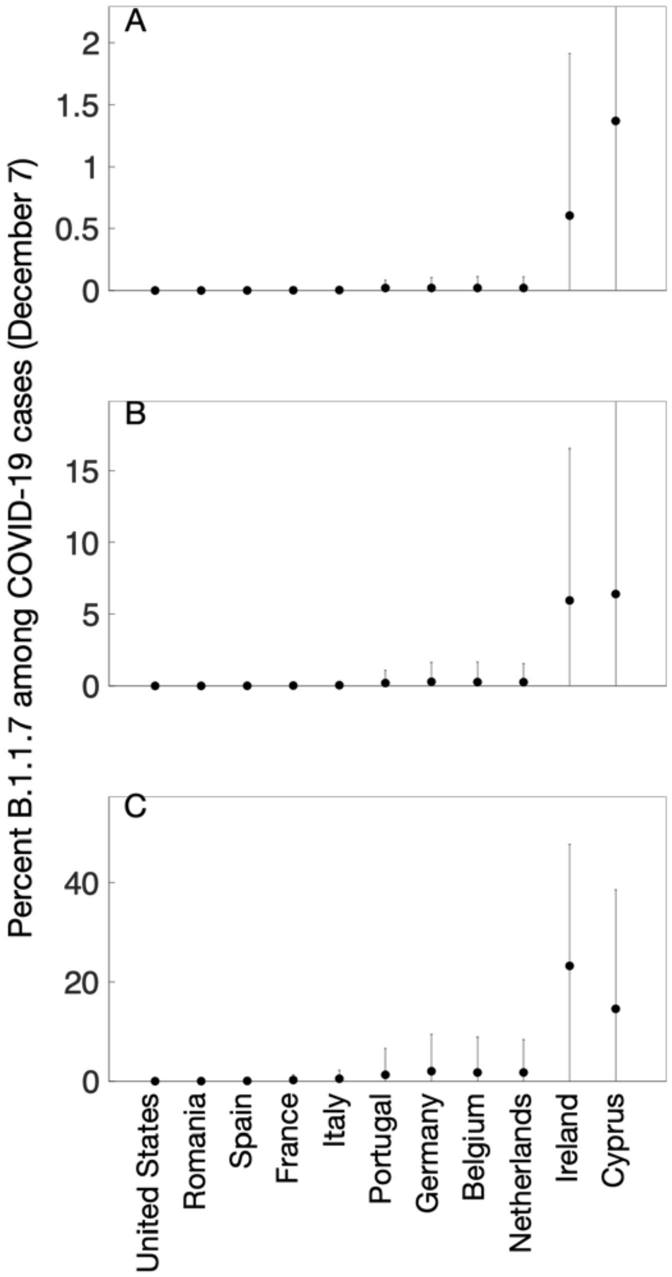

Appendix Figure 2. Estimated proportion of the 501Y variant among all SARS-CoV-2 cases in 11 countries on December 7, 2020, assuming the variant is σ = (A) 25%, (B) 50%, and (C) 75% more transmissible than 501N. Points and bars indicate means and standard deviations across 100 simulations.

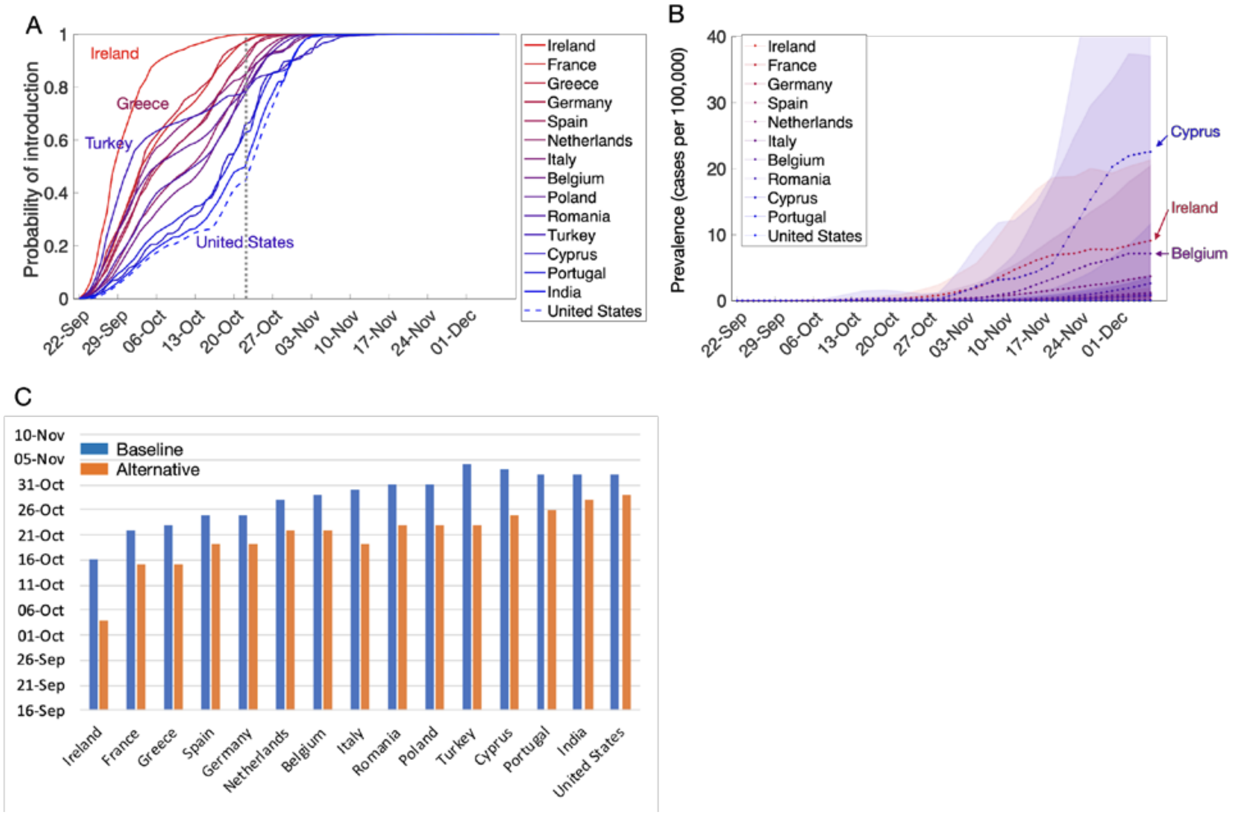

Appendix Figure 3. Sensitivity analysis assuming that symptomatic cases can travel and increasing the mobility flows by a factor of 1.8. (A) The probability that at least one person infected with the 501Y SARS-CoV-2 variant has arrived at the target country from the UK by the date indicated on the x-axis, based on Facebook mobility data. The dotted gray vertical line indicates October 23; line colors correspond to the relative risk of importations as of that date. (B) Estimated daily prevalence of the 501Y variant of SARS-CoV-2 in 11 countries between September 22 and December 7, 2020, assuming that the variant is σ = 50% more transmissible than the 501N variant (Davies NG, unpub. data, https://www.medrxiv.org/content/10.1101/2020.12.24.20248822v1.full-text)). Points and shaded bands indicate means and standard deviations based on 100 simulations. (C) The calendar day that the probability of a 501Y variant introduction first exceeds 0.8, for the baseline (blue) versus alternative (red) assumptions of elevated mobility and symptomatic travel.
